# Removal of High Concentrations of Ammonium from Groundwater in a Pilot-Scale System through Aeration at the Bottom Layer of a Chemical Catalytic Oxidation Filter

**DOI:** 10.3390/ijerph16203989

**Published:** 2019-10-18

**Authors:** Wushou Zhang, Ruifeng Zhang, Yanfeng Yang, Tinglin Huang, Gang Wen

**Affiliations:** 1 Key Laboratory of Northwest Water Resource, Environment and Ecology, MOE, Xi’an University of Architecture and Technology, Xi’an 710055, China; wushou-qingsheng@163.com (W.Z.); yangyanfeng0920@163.com (Y.Y.); 2 Shaanxi Key Laboratory of Environmental Engineering, Xi’an University of Architecture and Technology, Xi’an 710055, China; 3 School of Urban Planning and municipal engineering, Xi’an Polytechnic University, Xi’an 710048, China; ruifengzhangtry@163.com

**Keywords:** drinking water, ammonium, filtration, chemical catalytic oxidation

## Abstract

To remove high concentrations of ammonium from groundwater, pure oxygen and compressed air were fed into a chemical catalytic filter and the ammonium removal efficiency was investigated. The experimental results showed that the oxygen content is the critical limiting factor for ammonium removal. Aeration with 40 mL/min pure oxygen or 100 mL/min compressed air from the bottom of the filter supplied adequate oxygen and approximately 4.2 mg/L of ammonium was removed in this process. Moreover, when the aeration device was moved to 1/3 of the height of the filter bed, the required flow rates of pure oxygen and compressed air decreased further and the turbidity removal was improved. Pouring ozone gas into the filter system, which can inactivate bacteria effectively, can also obtain the remarkable ammonium removal, indicating that ammonium removal was mainly due to the chemical catalytic oxidation in this process rather than the biodegradation. This study provides a novel method for removing high concentrations of ammonium from groundwater.

## 1. Introduction

Ammonium is one of the major pollutants found in water sources in China, especially in groundwater [1]. Excessive ammonium in drinking water can cause nitrification in the water distribution system, leading to many problems including corrosion, aesthetic issues and pH decrease [2,3]. In China, the maximum permitted concentration of ammonium in drinking water is 0.5 mg/L. Therefore, it is meaningful to explore various techniques that can efficiently remove ammonium from drinking water.

Breakpoint chlorination is an effective way to remove ammonium from drinking water. It has low spatial requirement, non-sensitivity to temperature variations and adaptability to existing facilities. However, it can lead to high chlorine consumption and stimulate the formation of undesirable chlorinated by-products in the drinking water [4,5]. Biological filter and catalytic oxidation are good methods to remove ammonium from drinking water [6,7,8,9]. However, in biological filtration, the removal activity is limited at lower temperatures and the deficiency of some required nutrition [10,11,12]. Therefore, some measurements should be taken to improve the ammonium removal efficiency in biofiltration.

An alternative method to biofiltration would be abiotic removal of ammonium, using catalytic oxidation technology. Traditional catalytic oxidation processes can remove ammonia from municipal wastewater effectively using platinum and RuO_2_/Ti anode as catalysts [13,14,15]. However, these processes are used rarely in drinking water treatment systems. Recently, studies report that iron–manganese co-oxide filter film (MeO_x_) can remove ammonium from drinking water by chemical catalytic oxidation. It has good tolerance to low temperatures and requires a shorter start-up time compared to the conventional biofiltration method, making it a good alternative for ammonium removal from drinking water [6,8].

The application of chemical catalytic oxidation technology and its mechanism for ammonium removal has been studied, which showed that ammonium with an average concentration of approximately 1.5–2.0 mg/L could be removed effectively [7,8]. However, its effectiveness in removing high concentrations of ammonium has not been investigated. Moreover, the ammonium concentration in drinking water can be high in China. It can be up to 2–3.9 mg/L in ground water and 1.45–3.89 mg/L in surface water [9,16,17,18]. In some cases, it can reach up to 8.4 mg/L in raw water sources [19]. Therefore, exploring new methods to remove high concentrations of ammonium is significant to environmental engineering.

With respect to the low concentration of ammonium, high concentrations of ammonium are more difficult to remove based on the limitation of the inadequate dissolve oxygen (DO) concentration in water sources [19].

DO concentration is generally lower in groundwater than that in surface water and the DO limitation is more critical for ammonium removal [6,12,19,20]. On the one hand, the impact of oxygen transfer is a key factor that influences the ammonium removal and some previous researchers suggested that increasing irrigation velocity could increase the oxygen transfer for satisfying the metabolic requirements of both heterotrophs and autotrophs [21,22]. On the other hand, the required oxygen was usually supplied by multi-stage water-dropping and spray aeration in drinking water treatment processes when the concentration of ammonium was not too high [6,20]. However, the aeration method mentioned above cannot supply adequate oxygen to meet the required levels of ammonium oxidation, especially if the ammonium concentration is higher than 2 mg/L. Consequently, exploring new methods to provide adequate oxygen to remove high concentrations of ammonium is significant.

Different from the traditional multi-stage water-dropping and spray aeration, aeration with pure oxygen and compressed air via a microporous aeration set was used in this study to establish a new way for removing high concentrations of ammonium from groundwater by chemical catalytic oxidation filter. The effects of aeration types, aeration rates, and aeration position on ammonium or turbidity removal were investigated. Moreover, ozone, which can inactivate bacteria effectively, was also fed into the filter system to investigate the role of the abiotic chemical catalytic oxidation and the characteristics of the filter materials were examined to explore the removal mechanism of high concentrations of ammonium.

## 2. Materials and Methods

### 2.1. Pilot-Scale Filter System

**Figure 1:**
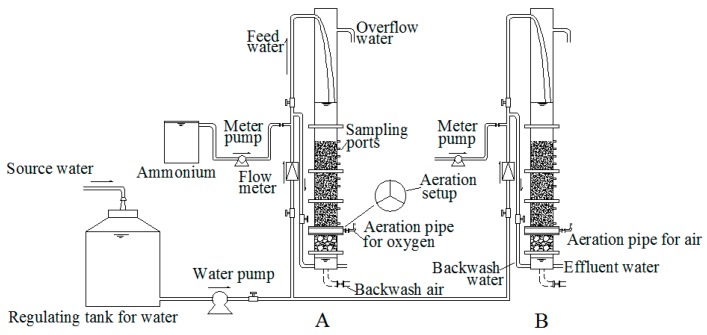
Schematic of the pilot-scale filter.

The pilot-scale filter system is shown in Figure 1. It consists of two identical filter columns and auxiliary equipment such as dosing pumps and aeration devices. The filter columns were made of plexiglass tubes with an inner diameter of 100 mm and a height of approximately 3.5 m. Quartz sand coated with iron–manganese co-oxide filter film (MeO_x_) for the chemical catalytic oxidation of ammonium was packed as the filter media, with an effective height of 1.2 m. The diameter of the filter was 1~2mm. The supporting layer consisted of 300 mm pebbles with a particle size of 4–8 mm. The maximum permitted head loss was 1.6 m. Compressed air or pure oxygen was dosed into the filter column by an aeration setup that consisted of three stainless steel tubes with microspores of approximately 10 μm. The installation site of the aeration setup can be adjusted by connecting it with a flange plate. Six sampling ports were settled at 10, 20, 30, 50, 60, 100 and 120 cm along the filter depth. NH_4_Cl stock solution was dosed into the source water to model the polluted groundwater. The operational time was one week for each experimental condition. The filtration cycle was approximately 24–48 h. Except special instructions, the filtration rate in this study was 8 m/h. The quality of the raw water (groundwater) used in this experiment was provided in Table 1. In this study, iron and manganese were removed easily and the removal efficiency was not influenced significantly by the aeration. Therefore, the removal of iron and manganese is not discussed yet.

### 2.2. Effects of Aeration Types

Two different aeration types were used to investigate the feasibility of the removal of high concentrations of ammonium. In one experiment, pure oxygen and compressed air were fed into the chemical catalytic oxidation filter system from the bottom layer by the special aeration devices as described above. In another experiment, oxygen was supplied by water-dropping aeration. The ammonium removal efficiency of the filter with different aeration types was investigated.

### 2.3. Effects of the Aeration Rates and Aeration Position

This experiment was designed to explore the relationship between aeration rates, aeration position and the ammonium removal efficiency. Firstly, the aeration rates of pure oxygen and compressed air were controlled at 30~50 mL/min and 75~150 mL/min from the bottom, respectively. After that, the aeration device was moved from the bottom to a position that was 1/3 of the height of the filter bed. The required aeration rates and removal efficiency of turbidity were investigated. 

### 2.4. Effects of Filtration Rates

The average filtration rate in other experiments was 8 m/h. In this experiment, filtration rates were set as 8 m/h, 10 m/h, and 12 m/h and the ammonium removal efficiency was investigated. Each operational condition lasted for one week.

### 2.5. Mechanism of Ammonium Removal

To study the removal mechanism of ammonium, the characteristics of the filter materials used in both the aeration processes were examined. Scanning Electron Microscopy (SEM, ESCALAB 250Xi) was used to investigate its morphology and energy dispersive spectrometer (EDS) was used to investigate its element composition. The specific surface area and pore properties of MeOx were evaluated using a surface area analyzer (BET) (ASAP 2020, Micromeritics Co., Beijing, China). In addition, ozone was also diffused into the filter from bottom in order to explore the changes in the ammonium removal efficiency.

### 2.6. Analytical Methods

The concentrations of ammonium, nitrite and nitrate were determined following the Chinese National Standard Methods [23]. pH and the DO concentration were measured using a portable instrument (HQ30d, HACH, Loveland, CO, USA).

## 3. Results and Discussion

### 3.1. Effects of Aeration Types

**Figure 2:**
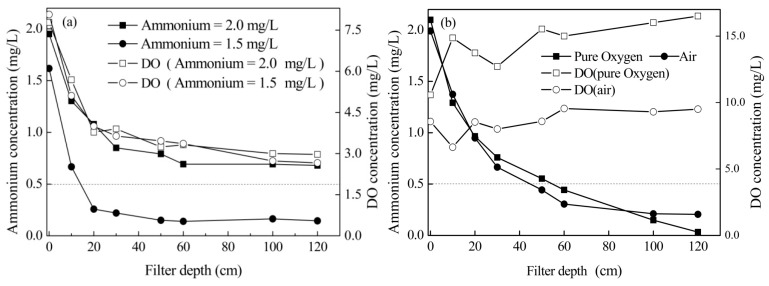
(**a**) Ammonium removal with aeration by water-dropping; (**b**) Ammonium removal with aeration using compressed air and pure oxygen from the bottom of the filter.

The ammonium removal efficiency of the filter with aeration by water-dropping is shown in Figure 2a. It indicated that ammonium can be removed effectively when its influent concentration was approximately 1.5 mg/L. However, the effluent ammonium concentration was higher than 0.5 mg/L when the influent concentration was approximately 2.0 mg/L. Although the influent ammonium concentrations were different, DO concentration depth profiles were almost identical in both the procedures. DO concentration at a filter depth of 100–120 cm was 2.66–3.00 mg/L. It indicated that an oxygen concentration higher than 2.66–3.00 mg/L was required for efficiently removing ammonium in a chemical catalytic filter. In normal biofiltration, the DO suggested concentration was 2.0 mg/L [24].

Theoretically, to oxidize 1 mg of NH_4_^+^-N, 4.57 mg/L of O_2_ will be consumed. Therefore, the insufficient DO could be the limiting factor for the removal of high concentrations of ammonium. Using water-dropping aeration, the DO in groundwater could be up to 6–7 mg/L [6,7]. Furthermore, using some special aerators, the concentration could be higher than 9 mg/L [20]. For surface water, the DO concentration fluctuated with seasonal variations and was in the range 6.5–12 mg/L [8,12]. The DO level in these water sources can meet the requirement for the oxidation of ammonium with a concentration of 1.31–2.63 mg/L theoretically. In this study, ammonium with a concentration higher than 2 mg/L was suggested as a high concentration.

To supply adequate oxygen for ammonium oxidation, compressed air and pure oxygen were supplied into the filter column from the bottom of the filter bed. The removal efficiency is shown in Figure 2b. It indicated that after aeration with pure oxygen and compressed air, the DO concentration in the filter system increased up to 10.57~16.51 mg/L and 6.64~8.57 mg/L, respectively. Meanwhile, the corresponding effluent ammonium concentration decreased to less than 0.5 mg/L. It showed that the DO concentration was the critical limiting factor for ammonium removal and when adequate oxygen was provided, a chemical catalytic oxidation filter could remove high concentrations of ammonium effectively.

### 3.2. Effects of Aeration Rate

**Figure 3:**
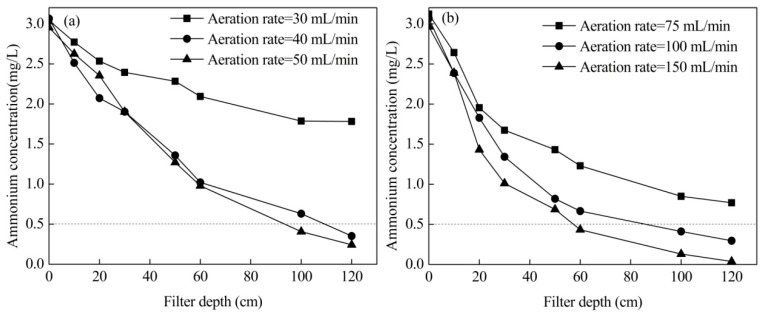
Ammonium removal with different aeration intensities: (**a**) pure oxygen and (**b**) compressed air.

The performance of the filter under different aeration intensities is demonstrated in Figure 3. It shows that when aeration with 40 mL/min of pure oxygen, the ammonium concentration could be reduced to less than 0.5 mg/L, while 100 mL/min of compressed air was required to obtain a similar performance. The DO concentration depth profiles in the filter at different aeration intensities (Figure S1) show that when the flow rates of pure oxygen and compressed air were 30 mL/min and 75 mL/min, respectively, the DO concentrations were much lower than that higher aeration intensity. Therefore, ammonium could not be removed effectively with these aeration intensities.

When compressed air aeration rate was 100 mL/min, the DO concentration decreased obviously at 0~20 cm filter depth and, after that, it increased with the filter depth. On the other hand, for aeration with pure oxygen at 40 mL/min, just a slight decrease in the DO concentration was detected at 0~20 cm filter depth and a high DO concentration was sustained in the whole filter bed. It suggests that the supplying oxygen capability of the 40 mL/min pure oxygen was higher than 100 mL/min compressed air and even approximate to 150 mL/min compressed air. However, the ammonium removal efficiency with 40 mL/min pure oxygen was slightly lower than that with compressed air under 100 mL/min. The reason for this may be that the higher gas flow rates can promote ammonium and oxygen transfer to the surface of iron–manganese co-oxide filter film coated on the filter sands. These results were similar to the phenomenon in biofilters [21,22].

### 3.3. Effects of Filtration Velocity

**Figure 4:**
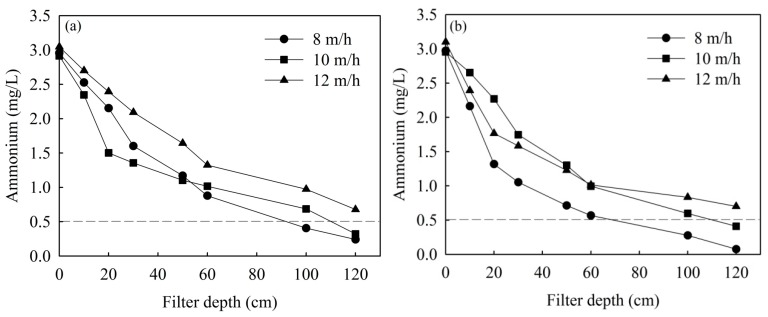
Ammonium removal with different filtration velocities: (**a**) pure oxygen and (**b**) compressed air.

The ammonium removal efficiency with different filtration velocities is shown in Figure 4. The ammonium concentration could be reduced to less than 0.5 mg/L when the filtration velocity was lower than 10 m/h when aerated with pure oxygen as well as compressed air. In general, the filtration velocity of the trickling filter and biological aerated filter (BAF) is 2.2–10 m/h [25,26,27] and 3.56–6.4 m/h [9,28], respectively. This indicated that chemical catalytic filters have some advantages over the traditional biofilter in operational loading rates.

### 3.4. Effects of Aeration on the Turbidity of the Effluent Water

**Figure 5:**
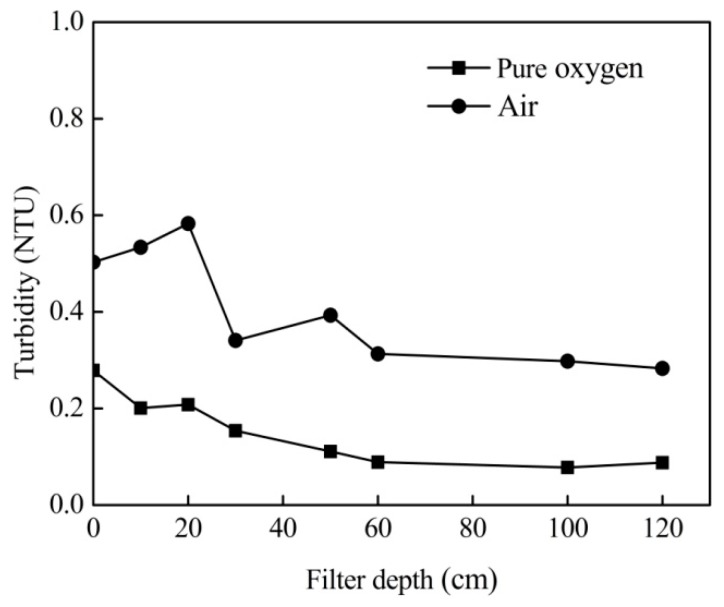
Concentration depth profiles of turbidity with aeration (40 mL/min pure oxygen and 100 mL/min compressed air).

Variations in the turbidity depth profiles with different aeration methods are shown in Figure 5. These suggests that turbidity was removed effectively with the aeration of pure oxygen as well as compressed air. However, it could be better removed with the pure oxygen aeration than that with the air aeration. The reason was attributed to the fact that the required aeration rate of pure oxygen was much lower than compressed air (i.e., 40 mL/min and 100 mL/min, respectively, in this study).

### 3.5. Effects of Aeration Position

**Figure 6:**
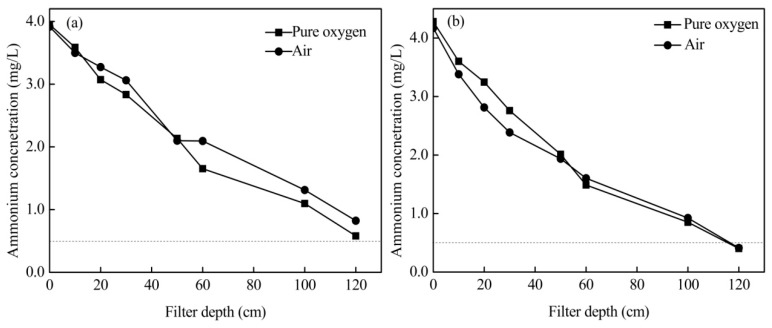
Ammonium removal efficiency with aeration at different positions: (**a**) aeration from the bottom and (**b**) aeration from 1/3 of the height of the filter bed (40 mL/min pure oxygen and 100 mL/min compressed air).

Aeration position plays a critical role in ammonium removal. Figure 6 gives the ammonium removal efficiency at different aeration positions. When aerating from the bottom of the filter bed, ammonium (approximately 4 mg/L) could not be removed to less than 0.5 mg/L. However, when the aeration position was moved to 1/3 of the height of the filter bed, ammonium could be removed efficiently to meet the drinking water standards in China. DO concentration profiles with aeration at 1/3 of the height of the filter bed (Figure S2) are obviously different from the profiles with aeration from the bottom of the filter bed (Figure S1). The DO concentration in this process increased with filter depth (7.96–16.75 mg/L, with pure oxygen) or was sustained at a high level in the whole filter bed (7.45–8.8 mg/L, with compressed air). This indicated that aeration at 1/3 of the height of the filter bed has more efficiency than aeration from the bottom. In the filter system, ammonium concentration was higher in the upper part of the filter bed. Therefore, more oxygen was required. When the aeration position was moved to 1/3 of the height of the filter bed, the whole interaction time of the gas with water decreased. However, more oxygen could to be provided to strengthen ammonium removal in the upper part of the filter bed. This conclusion can be drawn from the difference in the oxygen concentration in the 0–60 cm filter bed with different aeration positions (Figures S1 and S2).

### 3.6. Ammonium Removal Mechanism

To study the mechanism of ammonium removal when aerated with pure oxygen and compressed air, the morphology, element composition and specific area of the iron–manganese co-oxide filter film were examined.

**Figure 7:**
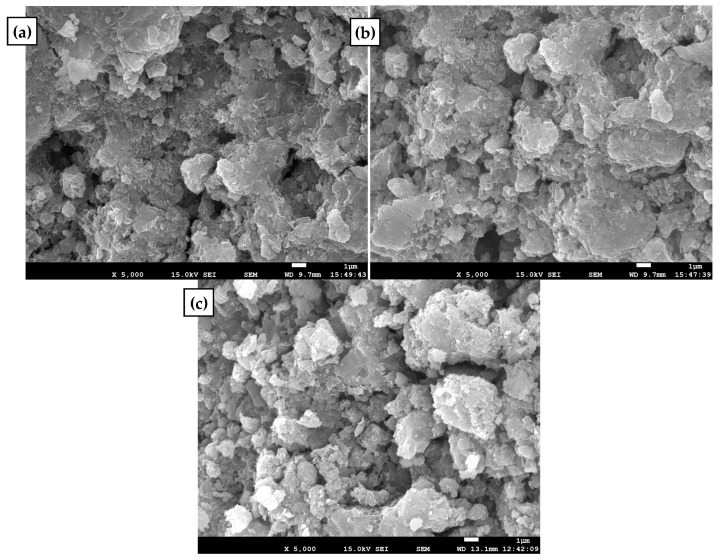
Scanning Electron Microscopy (SEM) images of the filter materials: (**a**) original, (**b**) aeration with pure oxygen, and (**c**) aeration with compressed air.

The experimental results showed that the morphology of the iron–manganese co-oxide filter film in both the aeration cases and the original was almost the same (Figure 7) and was porous and sponge-like. The major elements composition of MeO_x_ is shown in Table 2. It indicated that after aeration with pure oxygen and compressed air, the major elements in MeO_x_ are almost the same as those in the original MeO_x_. The specific surface area of the MeO_x_-coated sand is shown in Table S1. It shows that the specific area and pore characteristic of the MeO_x_-coated sand do not obviously change after aeration with pure oxygen and compressed air. These unchanged characteristics indicate that pure oxygen and compressed air improved ammonium removal by just providing adequate oxygen and did not change the mechanism of ammonium removal. Ozone experiments showed that fed ozone into the filter from the bottom of the filter bed could also improve ammonium removal just like pure oxygen and compressed air. On the one hand, ozone poured into the filter can transfer to be oxygen, which improved the ammonium removal efficiency. On the other hand, ozone can inactivate bacteria effectively (Figure S3). Moreover, it can be seen from Table S2 that the increase in nitrate concentration was similar to the concentration of the removed ammonium (the concentration of nitrite was relatively lower and was ignored). Therefore, the removed ammonium should be mainly oxidized by the chemical catalytic oxidation rather than be adsorbed. This conclusion was consistent with the results of previous study [6]. In the present study, two mechanisms have been proposed for the catalytic oxidation reaction and these were summarized [8]. One possible mechanism is that reactive oxygen species are formed that oxidize NH_4_^+^ to NO_3_^−^ when oxygen is adsorbed on the MnO_x_ surface [6]. Another possible mechanism is that NH_4_^+^ is oxidized directly by Mn(IV) or Mn(III) present in the filter film, resulting in the formation of NO_3_^−^. In this process, Mn(II) would be produced, followed by oxidation via self-catalyzed oxidation by MnO_x_ [7].

### 3.7. Comparison with Other Studies

Generally, trickling filters and biological aerated filters (BAFs) are used to remove high concentrations of ammonium from drinking water [5,25,27,29]. The experimental results from some of the relevant studies are summarized in Table 3.

A biofiltration unit at a pressure of 2 bars was used for groundwater treatment in a previous study. Although the concentration of oxygen in this system was up to 16–17 mg/L and efficiently removed the ammonium, a closed biofilter with stainless steel was required [30] and the construction cost of this system is high. 

The size of the filter materials in trickling filters is usually larger than that in GAC (granular activated carbon) filters and rapid sand filters [26,30,32] and water in trickling filters was partially filled pipe-flow to ensure adequate oxygen can be supplied. There are some drawbacks in trickling filter treatment process—for example, the rotating distributor for water distribution can cause slower water flow rates, clogging problems, and easily hit filter media [33] and a large area is needed to construct the treatment system. In BAFs, oxygen is supplied by aeration from the bottom of the filter. The removal efficiency is decided by the influent ammonium concentration and aeration rates [31,33]. Furthermore, as typical biological treatment processes, the removal efficiency of both the trickling filter and BAF were influenced by the nutrition, organic carbon loading and temperature [9,34]. To ensure the ammonium removal efficiency, the filter depth in BAFs and trickling filters should be high—up to 2.4–3.0 m [9,27]—which may also increase the construction cost. Moreover, limited by the biological risk and the turbidity of the effluent water, these treatment processes are usually used as pretreatment technologies in the whole drinking water treatment flow [9,27].

The chemical catalytic oxidation filter system can remove high concentrations of ammonium from drinking water effectively (Figure 3, Figure 4 and Figure 6). Additionally, it can also efficiently reduce water turbidity (Figure 5). Therefore, it can be used as the end treatment process, especially when the aeration point was transferred to 1/3 of the height of the filter bed. Furthermore, ammonium removal by chemical catalytic oxidation may have a lower risk of biological contamination compared to the traditional biological process.

## 4. Conclusions

This study investigated the efficiency of chemical catalytic oxidation filter systems to remove high concentrations of ammonium from drinking water using a pilot-scale filter system. The following conclusions were drawn:

(1) The removal efficiency is mainly limited by the content of dissolved oxygen in treated water. By aeration with pure oxygen and compressed air, high concentrations of ammonium can be removed effectively, and the suggested operational flow rates were 100 mL/min compressed air and 40 mL/min pure oxygen.
(2) Compared with aeration from the bottom, aeration at a position of 1/3 of the height of the filter bed can lower the required aeration rates and strengthened turbidity removal.
(3) Pure oxygen and compressed air can provide adequate oxygen without changing the mechanism of ammonium removal. In this process, ammonium was removed mainly by chemical catalytic oxidation.

## Figures and Tables

**Table 1:** Water quality of the raw water (groundwater) used in the pilot-scale filter system.

Parameters	Units	Values	Standards for Drinking Water Quality China (GB5749-2006)
NH_4_^+^-N	(mg/L)	0.70–1.67	0.5
NO_3_^-^-N	(mg/L)	0.0–0.3	10
Water temperature	(°C)	17.0–22.0	/
pH	/	7.50–8.5	6.50–8.5
Turbidity	(NTU)	0.80–1.6	1
DO	(mg/L)	2.00–3.2	/
COD_Mn_	(mg/L)	1.14–1.47	3
Iron	(mg/L)	0.70–1.1	0.3
Manganese	(mg/L)	1.10–1.56	0.1

“NTU” means nephelometric turbidity unit.

**Table 2:** The major elements composition of MeO_x_.

Elements	wt%		
	Original	Aeration with Pure Oxygen	Aeration with Compressed Air
O	14.51	13.54	13.24
Al	1.12	0.72	0.72
Ca	5.21	6.14	4.92
Mn	74	76.4	77.01
Fe	5.16	3.2	4.11

**Table 3:** Comparison of ammonium removal efficiency between previous and current studies.

Treatment System	Type of Water	Filter Media Types	DO in Effluent (mg/L)	Aeration Types	Temperature (°C)	Ammonium in Influent (mg/L)	Removal Efficiency (%)
Pilot-scale filters [This study]	Ground water for drinking water	MeO_x_-coated on quartz sand	7.45~8.8	Aeration from the bottom and the 1/3 of the filter bed	17~22	4.2	95.23
Pilot plant with at a pressure of 2 bars [30]	Ground water for drinking water	Quartz sand	16–17	Aeration at a pressure of 2 bars	14.6	2.62	100
Pilot-scale trickling filters [26]	Surface water for Potable water	Gravel	7~8	Natural ventilation	20	2	77.3~100
Sequence Batch BAF system [31]	Surface water for Drinking water	Poly-propylene	4.68	Aeration form the bottom	/	9.8	98.4
				Of the filter			
Continuous flow full Scale BAF [9]	Surface water for Drinking water	Lava particles	>7	Aeration form the bottom of the filter	8.6~10.8	2.90 ± 0.96	77.52~92.62
GAC-sand dual-media biofilters [19]	Surface Water for drinking water	GAC and sand	1.4~7.2	By aeration tank	/	0.74~5.3	39.77~99.32

“GAC” means granular activated carbon.

## Supplementary Materials

The following are available online at https://www.mdpi.com/1660-4601/16/20/3989/s1, Figure S1: Dissolved oxygen (DO) concentration depth profiles (aeration from the bottom of the filter bed) with different intensities of aeration, Figure S2: The DO concentration depth profiles with aeration at 1/3 of the height of the filter bed, Figure S3: Ammonium removal efficiency with aeration using ozone, Table S1: The specific surface area of the MeO_x_ coated sand, Table S2: Concentration of NH_4_^+^ and NO_3_^-^ in aeration with air, pure oxygen and ozone.
