# Supplementary material for: Removal of High Concentrations of Ammonium from Groundwater in a Pilot-Scale System through Aeration at the Bottom Layer of a Chemical Catalytic Oxidation Filter
Source: Int J Environ Res Public Health. 2019 Oct 18;16(20):3989. doi: 10.3390/ijerph16203989 (PMC6843939; doi:10.3390/ijerph16203989)
Supplement: Supplementary file 1 [file ijerph-16-03989-s001.pdf]

Figures

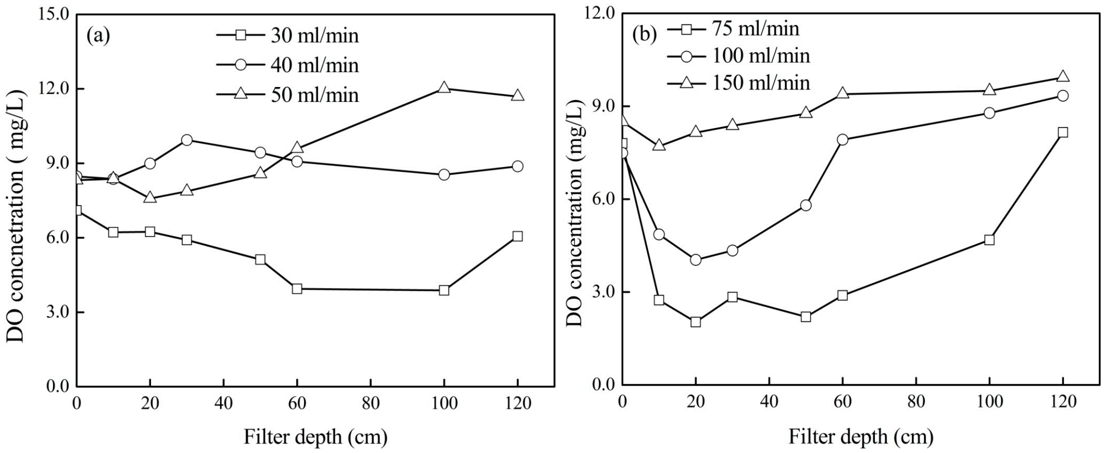

Fig.S1 Dissolved oxygen (DO) concentration depth profiles (aeration from the bottom of the filter bed) with different intensities of aeration: (a) Pure oxygen, (b) compressed air.

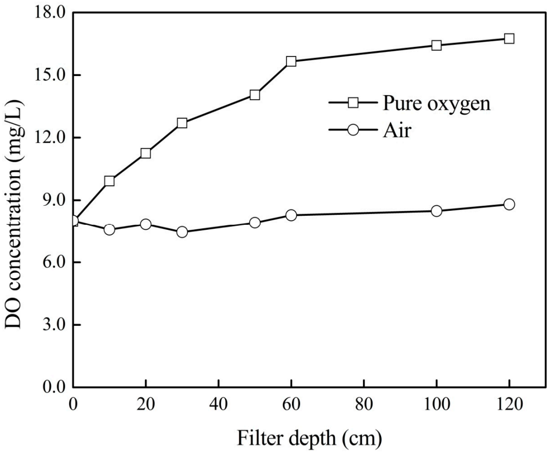

Fig.S2 The DO concentration depth profiles with aeration at 1/3 of the height of filter bed

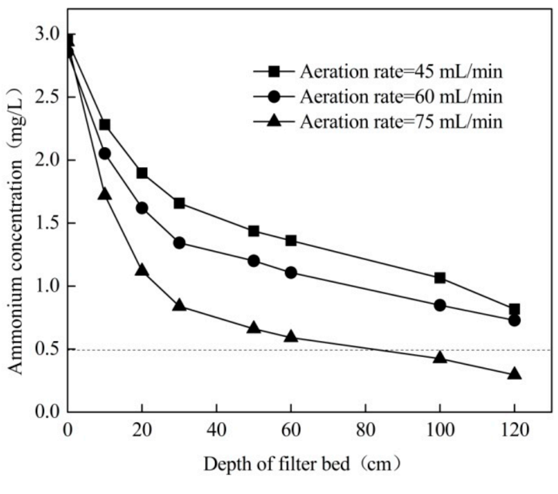

Fig.S3 Ammonium removal efficiency with aeration using ozone

Tables

Table S1 The specific surface area of the MeO_x coated sand.

filter material	Specific surface area（m^2/g）	pore volume（cm^3/g）	pore diameter（nm）
original	2.902	0.011	14.561
aeration with pure oxygen	2.814	0.010	14.461
aeration with compressed air	2.771	0.010	13.965

Table S2 Concentration of NH_4^+ and NO_3^- in aeration with air, pure oxygen and ozone

	Ammonium（mg/L）		Nitrate（mg/L）		The loss nitrogen(mg/L)
	Influent	Effluent	Influent	Effluent	
Air	3.155	0.323	0.092	2.797	0.127
Pure oxygen	3.055	0.242	0.105	2.876	0.042
Ozone	2.955	0.405	0.122	2.504	0.168
